# Draft Genome Sequence of NDM-1-Producing *Leclercia adecarboxylata*

**DOI:** 10.1128/genomeA.00135-17

**Published:** 2017-04-27

**Authors:** Yannick Hoyos-Mallecot, María Dolores Rojo-Martín, Rémy A. Bonnin, Elodie Creton, Jose María Navarro Marí, Thierry Naas

**Affiliations:** a Bacteriology-Hygiene Unit, Assistance Publique Hôpitaux de Paris (AP-PH), Bicêtre Hospital, Le Kremlin-Bicêtre, France; b Associated French National Reference Center for Antibiotic Resistance: Carbapenemase-Producing *Enterobacteriaceae*, Le Kremlin-Bicêtre, France; c EA7361 Structure, dynamic, function and expression of broad spectrum β-lactamases, Université Paris Sud, Université Paris-Saclay, LabEx LERMIT, Faculty of Medicine, Le Kremlin-Bicêtre, France; d Servicio de Microbiología Hospital Universitario Virgen de las Nieves, Granada, Spain; e Instituto Biosanitario de Granada, Granada, Spain; f Joint Research Unit EERA (Evolution and Ecology of Resistance to Antibiotics), Institut Pasteur, APHP, Université Paris Sud, Paris, France

## Abstract

Here, we provide the first draft genome sequence of NDM-1-producing *Leclercia adecarboxylata*, a human-opportunistic pathogen. The draft genome sequence consists of a total length of 5.13 Mbp, with an average G+C content of 55.2%.

## GENOME ANNOUNCEMENT

*Leclercia adecarboxylata* is a Gram-negative opportunistic pathogen mostly collected from environmental sources (1, 2). It may rarely be isolated from clinical samples, mostly from inmunocompromised patients, but rare infections in immunocompetent patients have also been described, however, they are often associated with polymicrobial flora (1, 3, 4). Therefore, the real implication of *L. adecarboxylata* in human infections is still unclear. *L. adecarboxylata* was first described by Leclerc in 1962 (5), but at the time it was classified as part of the genus *Escherichia* and was therefore known as *Escherichia adecarboxylata*. This classification was based on close phenotypical testing results, especially in terms of biochemical profiles but also in terms of susceptibility testing.

Although *L. adecarboxylata* (like *E. coli*) is generally sensitive to almost all β**-**lactams, a large variety of β-lactamases have been described, including some carbapenemases, mostly metallo-β-lactamases (3, 6). The emergence of carbapenemases in environmental isolates is of great concern, since these strains could act as reservoirs for further dissemination.

Genomic DNA was extracted using the UltraClean microbial DNA isolation kit (Mo Bio Laboratories) from overnight cultures in LB agar (Bio-Rad, Marnes-la-Coquette, France). Genomic DNA quantification was performed using a Qubit fluorometer (Life Technologies, Carlsbad, CA) and adjusted to 0.2 ng/L. Library preparation was performed using the Nextera XT DNA sample preparation kit (Illumina, San Diego, CA). Sequencing was performed on an Illumina Next 500 sequencer with V 3 chemistry using 2 × 150-bp paired-end reads.

Illumina sequencing resulted in 2,106,374 reads of an average length of 149.4 nucleotides, giving a total of 314,690,675 nucleotides. These generated reads were assembled and computed using CLC Workbench version 9.5.1 (Qiagen, Les Ulis, France). The average length of the 214 contigs was 24,010-bp, and the total contig length was 5,138,172-bp. with an average G+C content of 55.2%. These contigs were further annotated using the RAST server (http://rast.nmpdr.org/). The RAST system predicted 4,646 coding sequences. These coding sequences include subsystems involved in essential metabolism of the bacteria: 251 coding sequences (CDSs) were predicted in cell wall and capsule synthesis, with 131 involved in virulence, disease, and defense; 44 involved in cell division and cell cycle; 177 involved in fatty acid and lipid metabolism; 178 involved in the stress response; and 277 and 237 involved in protein and RNA metabolism, respectively.

Here, we provided the first whole-genome sequence of NDM-1-producing *L. adecarboxylata*, isolated from a soft tissue infection. This isolate produced a clinically relevant carbapenemase as well as other genes involved in resistances.

### Accession number(s).

This whole-genome shotgun project has been deposited at DDBJ/ENA/GenBank under the accession no. MUFS00000000.
